# Cyber-Physical Vulnerability Assessment in Smart Grids Based on Multilayer Complex Networks

**DOI:** 10.3390/s21175826

**Published:** 2021-08-30

**Authors:** Monica Alonso, Jaime Turanzas, Hortensia Amaris, Angel T. Ledo

**Affiliations:** 1 Department of Electrical Engineering, University Carlos III of Madrid, 28911 Leganés, Madrid, Spain; monica.alonso@uc3m.es (M.A.); jaime.turanzas@alumnos.uc3m.es (J.T.); 2 Physical Safety Department, University Center of Guardia Civil (CUGC), 28300 Aranjuez, Madrid, Spain; aledo@alumno.uned.es; 3 International Doctoral School, UNED (National University of Distance Education), 28015 Madrid, Spain

**Keywords:** vulnerability, complex networks, smart grids, cyber-physical systems, robustness, scale-free graph, multilayer networks

## Abstract

In the last decade, the main attacks against smart grids have occurred in communication networks (ITs) causing the disconnection of physical equipment from power networks (OTs) and leading to electricity supply interruptions. To deal with the deficiencies presented in past studies, this paper addresses smart grids vulnerability assessment considering the smart grid as a cyber-physical heterogeneous interconnected system. The model of the cyber-physical system is composed of a physical power network model and the information and communication technology network model (ICT) both are interconnected and are interrelated by means of the communication and control equipment installed in the smart grid. This model highlights the hidden interdependencies between power and ICT networks and contains the interaction between both systems. To mimic the real nature of smart grids, the interconnected heterogeneous model is based on multilayer complex network theory and scale-free graph, where there is a one-to-many relationship between cyber and physical assets. Multilayer complex network theory centrality indexes are used to determine the interconnected heterogeneous system set of nodes criticality. The proposed methodology, which includes measurement, communication, and control equipment, has been tested on a standardized power network that is interconnected to the ICT network. Results demonstrate the model’s effectiveness in detecting vulnerabilities in the interdependent cyber-physical system compared to traditional vulnerability assessments applied to power networks (OT).

## 1. Introduction

Smart grids are characterized by high digitisation that facilitates modernisation in electrical network infrastructure, active grid management, control and real-time communication with consumers, prosumers, and DSOs [1]. Smart grid digitisation has created new areas of work, in which the electrical and communications infrastructures are neither independent nor operate in isolation [2,3]. Consequently, both infrastructures must be integrated into a single cyber-physical system (CPS) that must be analysed holistically to identify potential vulnerabilities that may affect the security and continuity of the power supply.

The vulnerability of an electrical network can be defined as its ability to maintain stable operation under the loss or disconnection of an element in the power network [4,5]. Vulnerability analysis allows power network operators to improve the robustness of critical elements in the network and develop countermeasures against untimely failures [6]. Therefore, recognising the most vulnerable elements in a power grid will result in better operation [1,7] and provide an indispensable tool for DSO decision-makers [8].

Traditional vulnerability analysis has focused on determining weaknesses in the electrical infrastructure (OT) when an element is lost. This is known as structural vulnerability [8]. Several authors have used power flow techniques to determine the most vulnerable nodes in a network [9]. However, methods that employ DC power flow are not suitable for analysing cascading events, and AC power flow methods have high computational costs [8]. Other studies have employed topological methods that can define links between the structure and electrical characteristics of OT networks. The main advantages of topological methods are scalability, low computational costs, the ease of defining vulnerability indices, and the possibility of incorporating electrical information, such as line impedance, into models as weighted graphs [8]. Topological methods are functional, logical, and numerical. However, they have several limitations. Functional methods [9] are unsuitable for large power networks, logical methods [10] cannot be applied to cyber-physical systems, and numerical methods [11] have been applied to only small-scale networks.

In recent years, complex network theory has been used to analyse vulnerabilities in OT power networks [8,12,13,14,15,16]. Complex network indices, such as betweenness and net-ability, have been used to assess OT network vulnerabilities [15,17]. The work presented by [18] proposes an electrical network model, based on complex network theory, to identify lines that would affect the OT network’s robustness in the event of a loss or disconnection. The authors of [16] used an adjacent graph model, and in [19], complex network indices, such as node degree and geodesic distance, were used to establish the critical nodes in an OT network that could cause a blackout in the event of cascading failures in specific elements. It should be noted that previous research papers have focused on physical electricity networks and have not addressed vulnerabilities that could occur in IT communications networks producing OT network vulnerabilities.

Current smart grids are cyber-physical systems composed of two heterogeneous networks: the power (OT) and communications (IT) networks which are both interconnected. Consequently, a vulnerability in either network (OT-IT) affects the entire CPS [3,20]. The work discussed in [21] represents an initial approach to analysing vulnerabilities in smart grids by considering them as a single CPS. The authors of [21] propose the use of an adjacency matrix, based on complex numbers, in which the OT network is represented by real components and the IT network by imaginary elements. However, the analysis developed in [21] determines the vulnerability of individual elements in isolated networks, but it does not perform a vulnerability analysis that detects the most critical node in the CPS as a whole.

To identify smart grid vulnerabilities, it is not sufficient to separately analyse the physical components and the computational components, as is assumed in [3]. It is also necessary to model the interaction between them. In [12,13,14,15,16,17,18,19], traditional vulnerability assessment is only based on the power grid; however, the detection of vulnerabilities in power networks is not sufficient to analyse vulnerabilities in smart grids which are also composed of communication networks. Moreover, to highlight hidden interdependencies between power and ICT networks it is also necessary to include the interconnection links between power nodes and communication nodes. 

The major novelty and contribution of our paper is the proposal of a new methodology based on a multilayer network for the analysis of vulnerabilities of the smart grid. In this paper, we model a smart grid as a whole cyber-physical system composed of two heterogeneous interconnected networks: power network, ICT network, and also the coupling network between both networks. This approach has not been considered in other publications as can be seen in Table 1.

It can be concluded through this comparison that interdependencies between power and ICT networks in smart grids are not considered in the literature [12,13,14,15,16,17,18,19]. These interdependencies represent one of the challenges in smart grids modelling [3]. This research field is in its early stage [21] and our proposed coupled smart grid modelled represents a contribution to the field.

In this article we propose the following three-fold contribution:

− We present an interconnected heterogeneous smart grid model for vulnerability assessment that highlights hidden interdependencies between power and ICT networks. The interconnected model encompasses power networks, ICT networks, and the interconnection between power-ICT networks. It should be emphasized that the interconnected and heterogeneous nature of smart grids is not considered in traditional smart grids vulnerability assessment where only power network vulnerability is analysed, and neither ICT network nor power-ICT interconnection is considered [12,13,14,15,16,17,18,19].
− We use multilayer complex network theory to deal with the complexity and heterogeneity of cyber-physical systems considered as interconnected and heterogeneous systems. Traditional smart grids models are modelled as monolayer networks in which only power network is considered [12,13,14,15,16,17,18,19,21], therefore they are not able to detect ICT vulnerabilities nor the interaction between power and ICT networks.
− To determine the vulnerabilities of the cyber-physical systems, we use multilayer complex network centrality indexes which allow us to detect hidden interdependencies between power and ICT systems. These interdependencies are not considered in monolayer centrality indexes [12,13,14,15,16,17,18,19,21] because they do not consider the interconnection between power and ICT networks.

The content of the paper is organized as follows: Section 2 introduces the multilayer network theory and their centrality indexes used for vulnerability assessment in multilayer networks. In Section 3, a Cyber-physical smart grid model is presented which is composed of two interconnected heterogeneous networks (power network, ICT network) and by the coupling layer between both networks. In Section 4, the proposed methodology is applied to a standardized power network where the communication network and communication components are included. Finally, the conclusions of the paper are detailed in Section 5.

## 2. Multilayer Theory

Complex network theory is one of the most widely used tools for analysing large numbers of interconnected elements, an area that has presented significant modelling challenges in recent years [22]. This theory has been used to represent networks in the fields of biology and sociology. It is also valid for representing power networks [17]. Complex network theory has been used to determine the most critical nodes in an electrical grid. In [23], the topology of an Iranian power network was modelled using complex network theory which included electrical network characteristics, such as line admittance. The authors of [24] used complex networks to analyse the impacts of attacks on a model of the French power network. Similarly, complex network theory has allowed the authors of [25] to identify the sequence of events that would produce a blackout in a 25-bus electrical network. What these works have in common is that they analyse network vulnerabilities by considering only the OT infrastructure topology and exclude the physical monitoring and control equipment installed in generators, substations, lines, or consumers. Similarly, previous studies have failed to consider the communication equipment responsible for transmitting the bidirectional flow of information between the electricity grid and the DSO control centre.

In this article, we propose a grid model based on multilayer complex network theory, in which two heterogeneous networks: the OT and IT networks are integrated into a single CPS.

### 2.1. Basic Definitions

A multilayer network is composed of M layers represented by the pair ℳ={G,C}, where G represents the family of graphs corresponding to each layer of the network, which are expressed according to $G=\left\{G_{\alpha },\;\alpha \in \left\{1,\ldots ,\;M\right\}\right\}$. Each network layer is represented by a graph consisting of a set of nodes or vertices ($N^{\alpha }$) and a set of edges between nodes ($E^{\alpha }$) so that the graph corresponding to layer α is represented by $G_{\alpha }=\left(N^{\alpha },E^{\alpha }\right)$.

The matrix (C) of the pair M,$\;C=\left\{E_{\alpha \beta }\subseteq N^{\alpha }\times N^{\beta };\;\alpha ,\beta \in \left(1,\ldots ,\;M\right);\;\alpha \neq \beta ,\right\}$ represents the connectivity between the nodes in the multilayer system ($n^{\alpha }\in G_{\alpha },\;\;m^{\beta }\in G_{\beta },\alpha \neq \beta$). The elements that comprise the matrix (C) are termed “cross layers”. The links between nodes in the same layer ($E^{\alpha }$) are called “intralayers”, while the joins between different layers are called “interlayers”, $E^{\alpha \beta }\;\left(\alpha \neq \beta \right)$. Hence, the multilayer system (ℳ) is composed of N nodes, where $N=\underset{\alpha }{\sum }N^{\alpha },\;\alpha \in \left\{1,\ldots .,\;M\right\}$ and $N^{\alpha }=\left\{n_{1}^{\alpha }\ldots n_{N_{\alpha }}^{\alpha }\right\}$.

According to layer α in the multilayer network, the graph $G_{\alpha }\;$can be represented by an adjacency matrix $A^{\left[\alpha \right]}=\left(a_{ij}^{\alpha }\right)\in {\mathbb{R}}^{N_{\alpha }\times N_{\alpha }}$, where each element of the adjacency matrix ($a_{ij}^{\alpha }$) is expressed as follows (1):

(1) $$a_{ij}^{\alpha }=\{\begin{array}{c} \begin{array}{cc} 1, & if\;\left(n_{i}^{\alpha },\;n_{j}^{\alpha }\right)\in E^{\alpha }\; \end{array}\\ \begin{array}{cc} 0,\;\;\;\;\;\;\;\;\; & otherwise \end{array} \end{array}\;\forall \;1\;\leq i,j\;\leq N^{\alpha },\;\;1\leq \alpha \leq M$$

Therefore, two nodes are considered to be adjacent in layer $\alpha \;(a_{ij}^{\alpha }=1)$ when an edge joins them ($E_{ij}^{\alpha }$). In multilayer systems, the concept of adjacency also extends to connections between nodes in different layers. Hence, the edge connecting the node-layer pairs $(n^{\alpha },\;\alpha )\;$and $(m^{\beta },\;\beta )\;$would be adjacent to node n in layer α and node m in layer β [26].

In multilayer systems, the adjacency matrix corresponding to the links between the *α* and *β* layers, denoted as$\;E^{\alpha \beta }$, is represented by $A^{\left[\alpha ,\beta \right]}=\left(a_{ij}^{\alpha \beta }\right)\in {\mathbb{R}}^{N_{\alpha }\times N_{\beta }}$, where the elements of the adjacency matrix are obtained using the following Equation (2):

(2) $$a_{ij}^{\alpha \beta }=\{\begin{array}{c} \begin{array}{cc} 1, & if\;\left(n_{i}^{\alpha },\;n_{j}^{\beta }\right)\in E^{\alpha \beta }\; \end{array}\\ \begin{array}{cc} 0,\;\;\;\;\;\;\;\;\; & otherwise \end{array} \end{array}\;\forall \;1\;\leq i,j\;\leq N^{\alpha },\;\;1\leq \alpha \leq M$$

Representing complex systems in layers allows us to model the nodes, the relationships between nodes in the same layer and connections between elements in different layers [22]. 

In multilayer systems, nodes in different layers are related to each other (i.e., nodes in one layer may depend on control nodes in other layers). Dependencies among nodes in different layers result in a structure known as a mesostructure in the field of complexity sciences. The mesostructure relates a node $n^{\alpha }\in G_{\alpha }$ with one or more nodes$\;n^{\beta }\in G_{\beta },\;1\leq \beta \leq M,\;\alpha \neq \beta$. It should be noted that such relationships between nodes are only possible in multilayer representations that allow the existence of the mesostructure.

In addition, multilayer systems can include nodes that are located in different layers. The connection or edges between nodes in different layers is called “coupling” ($\hat{C}$). An edge belongs to the coupling matrix if it links two nodes (n, m) that are present in two layers (*α* and *β*) (i.e., $E_{n,m}\;\in \hat{C}$ if$\;\;n^{\alpha }\in G_{\alpha },\;\;m^{\beta }\in G_{\beta },\;1\leq \alpha ,\beta \leq M,\;n=m,\;\alpha \neq \beta$). The nodes belonging to the coupling matrix are known as supra-nodes, and the graph formed by the supra-nodes and the coupling matrix is denoted by$\;{\hat{G}}_{C}$.

The supra-graph is the linked representation of the intra-layer and coupling graphs in a multiplex system. In multilayer systems with nodes in different layers, the connectivity matrix (C) is represented by $C=\left\{E^{\alpha \beta }\subseteq N^{\alpha }\times N^{\beta };\;\alpha ,\beta \in \left(1,\ldots ,\;M\right);\;\alpha \neq \beta \right\}\backslash \hat{C}$. 

### 2.2. Supra-Adjacency Matrix

A supra-adjacency matrix$\;({\bar{A}}_{ℳ}$) is defined as the adjacency matrix used to synthetically represent a multilayer graph (ℳ). Using the supra-adjacency matrix to represent multilayer systems allows us to use the tools and methods developed for monoplex systems.

The supra-adjacency matrix is obtained from the adjacency matrices corresponding to each layer and the connectivity matrix (C) between the different layers of the graph according to the following expression (3):

(3) $${\bar{A}}_{ℳ}={⊕}_{\alpha }A^{\left[\alpha \right]}+\;{⊕}_{\alpha ,\beta }A^{\left[\alpha ,\beta \right]}\;\;,\;\;1\leq \alpha ,\beta \leq M$$

where ${⊕}_{\alpha }A^{\left[\alpha \right]}$ is the intra-layer adjacency matrix and ${⊕}_{\alpha ,\beta }A^{\left[\alpha ,\beta \right]}$ is the inter-layer adjacency matrix corresponding to the connectivity matrix C.

**Figure 1:**
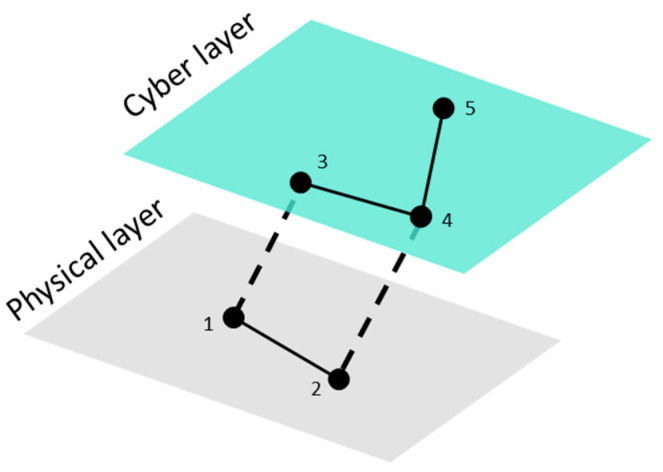
An example of a basic OT-IT multilayer system.

Figure 1 presents an example of a system with two layers; the physical layer contains two OT nodes (1, 2) and the cyber layer comprises three IT nodes (3, 4, and 5). The intra-layer adjacency matrices, corresponding to each layer as well as the intra-layer matrix, are defined by the matrices $A^{\left[1\right]}$ (4), $A^{\left[2\right]}$ (5), and ${⊕}_{\alpha }A^{\left[\alpha \right]}$ (6). Similarly, the connectivity matrix between the layers is expressed by C (7), and it is obtained from the inter-layer adjacency matrices $A^{\left[1,2\right]}$ (8), where$\;A^{\left[2,1\right]}=A^{\left[1,2\right]}{}^{T}$. Finally, the supra-adjacency matrix corresponding to the multilayer system in Figure 1 is represented by ${\bar{A}}_{ℳ}$ (9).

(4) $$A^{\left[1\right]}=\left[\begin{array}{cc} a_{1\_1} & a_{1\_2}\\ a_{2\_1} & a_{2\_2} \end{array}\right]$$

(5) $$A^{\left[2\right]}=\left[\begin{array}{ccc} a_{3\_3} & a_{3\_4} & a_{3\_5}\\ a_{4\_3} & a_{4\_4} & a_{4\_5}\\ a_{5\_3} & a_{5\_4} & a_{5\_5} \end{array}\right]$$

(6) $${⊕}_{\alpha }A^{\left[\alpha \right]}=\left[\begin{array}{cc} A^{\left[1\right]} & 0\\ 0 & A^{\left[2\right]} \end{array}\right]$$

(7) $$C={⊕}_{\alpha ,\beta }A^{\left[\alpha ,\beta \right]}=\left[\begin{array}{cc} 0 & A^{\left[1,2\right]}\\ A^{\left[2,1\right]} & 0 \end{array}\right]$$

(8) $$A^{\left[1,2\right]}=\left[\begin{array}{ccc} a_{1\_3} & a_{1\_4} & a_{1\_5}\\ a_{2\_3} & a_{2\_4} & a_{2\_5} \end{array}\right]=A^{\left[2,1\right]}{}^{T}$$

(9) $${\bar{A}}_{ℳ}=\left[\begin{array}{cc} A^{\left[1\right]} & A^{\left[1,2\right]}\\ A^{\left[2,1\right]} & A^{\left[2\right]} \end{array}\right]$$

### 2.3. Supra-Laplacian Matrix

The Laplacian matrix corresponding to an adjacency matrix is defined as follows (10):

(10) ℒ=D−A

where$\;D=diag\;\left(k_{1},\;\ldots ,\;k_{M}\right)$ is the array containing the degree index for each layer in the system.

In the case of multilayer systems, the Laplacian matrix is expressed as follows (11):

(11) $${\bar{ℒ}}_{ℳ}={\bar{D}}_{ℳ}-{\bar{A}}_{ℳ}$$

where ${\bar{D}}_{ℳ}=diag\;\left(K_{1},\;\ldots ,\;K_{M}\right)$, is a diagonal matrix that collects the degree index associated with the supra-adjacency matrix ${\bar{A}}_{ℳ}$.

From the Laplacian matrix in the multilayer system (${\bar{ℒ}}_{ℳ}$), it is possible to obtain the Laplacian matrices for each graph ($G_{\alpha }$) in the system, as well as the mesostructure represented by the connectivity matrix (C) using the following Equations (12) and (13):

(12) $${ℒ}^{\left[\alpha \right]}=D^{\left[\alpha \right]}-A^{\left[\alpha \right]}$$

(13) $${ℒ}_{C}=D^{\left[C\right]}-C$$

where $D^{\left[C\right]}=diag\left(c_{i}^{\left[1\right]},\;\ldots \;c_{i}^{\left[M\right]}\right)$.

From (13), we can define the supra-Laplacian matrix corresponding to a multilayer system (ℳ) using the following Equation (14):

(14) $${\bar{ℒ}}_{ℳ}={⊕}_{\alpha }{ℒ}^{\left[\alpha \right]}+{ℒ}_{C}$$

### 2.4. Multilayer Indexes for Vulnerability Assessment

Determining the most important nodes within a complex system, such as a multilayer network, is one of the main challenges in complexity sciences. Within the literature, several indices, known as centrality indexes, have been used to rank node vulnerability in the complete system. The main indexes used in multilayer systems are discussed in the following sections. 

#### 2.4.1. Topology-Based Indexes

Indexes related to the adjacency matrix (3) can be used to assess a network’s vulnerability. This group includes the following indices: node degree, closeness, and betweenness.

− The node degree index refers to the degree of centrality in a node, which measures the node’s level of connectivity with the remaining nodes in the system (in either single- or multilayer systems). Therefore, a node that is connected to many nodes will have a greater influence on the remaining nodes compared to those that have a smaller number of connections.
  The degree of centrality of a node,$\;i\;(i\equiv n^{\alpha }\in \;G_{\alpha }$), in the family of graphs belonging to the complex multilayer system ℳ(G,C), is calculated from the vector $k_{i}$ (15):
  (15) $$k_{i}=\left(k_{i}^{\left[1\right]},\;\ldots ,\;k_{i}^{\left[M\right]}\right)$$
  where $k_{i}^{\left[\alpha \right]}$ is the degree of centrality of node i in layer α, calculated according to$\;k_{i}^{\left[\alpha \right]}=\underset{j}{\sum }a_{ij}^{\left[\alpha \right]},\;1\leq \alpha \leq M$. It should be noted that (15) is not sufficient to evaluate the vulnerability of a node within a multilayer system (i.e., in$\;{\mathbb{R}}^{M})$. Therefore, the overlapping degree (hereafter node degree) is used. This is obtained by adding the information collected using the vector (15) for a node n∈ ℳ, in the following expression (16).
  (16) $$O_{i}=\sum_{\alpha =1}^{M}k_{i}^{\left[\alpha \right]}$$
− The closeness index quantifies a node’s vulnerability according to the shortest distance ($d_{ij}$) between the node and all remaining nodes. In general, the closeness of a node ($n_{i}^{\alpha }$) is calculated using the following Formula (17):
  (17) $$Closeness_{i}=\frac{1}{N^{\alpha }-1}\overset{N^{\alpha }}{\underset{\begin{array}{c} j=1\\ i\neq j \end{array}}{\sum }}\frac{1}{d_{ij}}\;i,j\in \;N^{\alpha }$$
− The betweenness index quantifies a node’s relevance by measuring the number of shortest paths from one node to the remaining nodes via the minimum number of links. If the betweenness value is high, this implies that the node is critical since the loss of that node reduces the network’s robustness. The betweenness index of a node ($n_{i}^{\alpha }$) can be calculated using the following Equation (18):
  (18) $$Betweenness_{i}=\frac{1}{\left(N^{\alpha }-1)(N^{\alpha }-2\right)}\overset{N^{\alpha }}{\underset{\begin{array}{c} j,s=1\\ i\neq j\neq s \end{array}}{\sum }}\frac{\sigma_{js}\left(i\right)}{\sigma_{js}}\;i,j\in \;N^{\alpha }$$
  where $\sigma_{js}\;$represents the shortest path between the nodes j and s, and$\;\sigma_{js}\left(i\right)$ is the number of paths containing or passing through the node i.

#### 2.4.2. Indexes Related to Laplacian Matrix

The centrality of a node$\;i\;(i\equiv n^{\alpha }\in \;G_{\alpha }$), $c\_eig_{i}^{\alpha }$, can be expressed by (19) from the spectral characteristics of the Laplacian matrix:

(19) $$\lambda c\_eig_{i}^{\alpha }=\overset{N^{\alpha }}{\underset{j=1}{\sum }}a_{ij}c\_eig_{j}^{\alpha }$$

where λ is a constant of proportionality and $a_{ij}$ represents the centrality of the node i as a function of its connection with adjacent nodes (i.e., the adjacency matrix). Expressing (19) in matrix format, the graph$\;G_{\alpha }$ is obtained using the following Equation (20):

(20) $$A^{\left[\alpha \right]}{}^{T}C\_eig=\lambda C\_eig$$

The eigenvector index is obtained from the norm of the eigenvector associated with the largest eigenvalue of [27].

According to the Perron–Frobenius theory, the eigenvalues (λ) and eigenvectors (C_eig) of the Laplacian matrix allow us to obtain the algebraic connectivity and the Fiedler vector represented by the second smallest eigenvalue and its associated eigenvector respectively. These indexes determine the subgraphs into which a network can be divided. Algebraic connectivity identifies the most vulnerable connections, as well as those connections that can lead to a network blackout if a sequence of cascade events is initiated.

## 3. Cyber-Physical Smart Grid Model

A smart grid is a complex CPS composed of an electrical network and a communications network. Both infrastructures are connected via the devices that link the physical equipment in the OT layer with the ICT devices in the IT layer [28]. Consequently, it is necessary to model each infrastructure (electricity and communication) and the connection between them.

### 3.1. Model of the OT Layer Corresponding to the Power Network

Electrical networks are usually represented by the adjacency matrix. Using complex network theory, the electrical network is represented by a graph: $G_{P}=\left\{N^{B},\;E^{B}\right\}$ where, $N^{B}$ is the set of electrical $\left(n^{B}\right)$ nodes, and $E^{B}$ includes the $\left(e^{B}\right)$ connections between them. The electrical network adjacency matrix is expressed as follows (21):

(21) $$A^{\left[B\right]}=a_{ij}^{p}\;\in \;{\mathbb{R}}^{n^{B}xn^{B}}$$

where $a_{ij}^{p}=1$ if there is an electrical connection between nodes, $i,j\;\in \left\{1,\ldots ,n^{B}\right\},$ and $a_{ij}^{p}=0$ if the nodes are not connected.

Traditional vulnerability analyses [21,23,24] consider only the electrical nodes in the $G_{B}$ network and the connections between them.

It is important to note that smart grids are formed of both electrical and communications infrastructures. Measurement and control equipment are installed in the electrical network, and these are linked to ICT devices (e.g., routers). In a smart grid, the consequences of cyberattacks range from altered measurements and control signals to the disconnection of power network elements, such as generators, lines, and loads. Therefore, a model of an electrical network layer must include not only electrical network elements (generators, loads, lines, and substations) but also measurement and control equipment (merging units, controllers and IEDs), which belong to the OT layer and the OT-IT bridge.

Hence, an electrical model of a smart grid must include the set of electrical nodes in the power network ($N^{B}$) and the set of measurement, protection and control devices corresponding to the OT-IT bridge ($N^{OT/IT}$). The extended electrical network ($N^{P}$) graph consists of$\;N^{P}=N^{B}\cup^{\;}N^{OT/IT}$.

**Figure 2:**
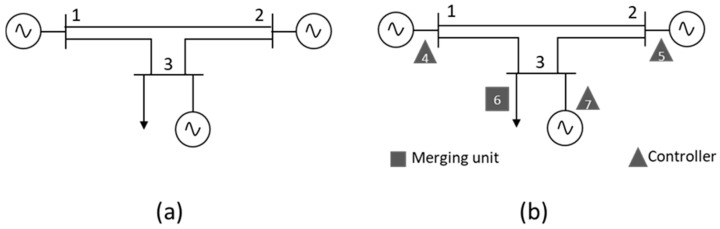
Three-bus smart grid representation with (**a**) electrical nodes and (**b**) electrical and OT-IT nodes.

Figure 2a shows an example of a three-bus smart grid, in which three generators (bus1, bus2 and bus3) and one load (bus3) are connected. The traditional electrical graph representing the network shown in Figure 2a is composed of three electrical nodes (set $N^{B}$ with $n^{B}=3)$. However, this smart grid also consists of three controllers, responsible for managing the generators, and a merging unit (MU) in the load that performs measurement tasks. The controllers and MU create set $N^{OT/IT}$ with $n^{OT/IT}=4$. Since the controllers and MUs are devices installed in the electrical infrastructure, they must be incorporated into the extended power grid graph. In this case, the three-bus OT network in Figure 2a is represented by an extended graph ($\hat{G_{P}}=\left\{N^{P},\;E^{P}\right\}$) comprising seven nodes ($n^{P}=n^{B}+n^{OT/IT};\;N^{P}=N^{B}\cup^{\;}N^{OT/IT}$) that correspond with the three electrical nodes, the three generator controllers and one load’s MU, as shown in Figure 2b.

The adjacency matrix of the graph corresponding to Figure 2a (set$\;N^{B}$) is given by $A^{\left[B\right]}$ (22) and has 3 × 3 ($n^{B}$ × $n^{B})$ dimensions. Incorporating OT-IT bridge elements into an extended model adds four nodes to the system (numbered from 4 to 7 in Figure 2b), which belong to set$\;N^{OT/IT}$. Matrix $A^{\left[OT/IT\right]}$ (23) represents the connection between the electrical nodes in the traditional network and the OT-IT bridge elements. The dimension of the array is therefore $A^{\left[OT/IT\right]}$ 3 × 4.

The adjacency matrix corresponding to the extended model $A^{\left[P\right]}$ (25) (Figure 2b) is obtained from the new set of nodes $N^{P}$. Matrix $A^{\left[P\right]}$ has dimension 7 × 7 and is composed of the following subarrays:

- $A^{\left[B\right]}$ (22) is the adjacency matrix corresponding to the traditional electrical network (nodes 1 to 3).
- $A^{\left[OT/IT\right]}$ (23) is the adjacency matrix representing the connection between the OT elements and OT-IT bridge (nodes 4 to 7), and their transpose ($\;A^{\left[OT/IT\right]}{}^{T}$).
- $A^{\left[bridge\right]}$ (24) is the connection matrix among bridge elements in the OT network.
  (22) $$A^{\left[B\right]}=\left[\begin{array}{ccc} a_{1\_1} & a_{1\_2} & a_{1\_3}\\ a_{2\_1} & a_{2\_2} & a_{2\_3}\\ a_{3\_1} & a_{3\_2} & a_{3\_3} \end{array}\right]$$
  (23) $$A^{\left[OT/IT\right]}=\left[\begin{array}{cccc} a_{1\_4} & a_{1\_5} & a_{1\_6} & a_{1\_7}\\ a_{2\_4} & a_{2\_5} & a_{2\_6} & a_{2\_7}\\ a_{3\_4} & a_{3\_5} & a_{3\_6} & a_{3\_7} \end{array}\right]$$
  (24) $$A^{\left[bridge\right]}=\left[\begin{array}{cccc} a_{4\_4} & a_{4\_5} & a_{4\_6} & a_{7\_7}\\ a_{5\_4} & a_{5\_5} & a_{5\_6} & a_{7\_7}\\ a_{5\_4} & a_{5\_5} & a_{6\_6} & a_{7\_7}\\ a_{5\_4} & a_{5\_5} & a_{5\_6} & a_{7\_7} \end{array}\right]$$
  (25) $$A^{\left[P\right]}=\left[\begin{array}{cc} A^{\left[B\right]} & A^{\left[OT/IT\right]}\\ A^{\left[OT/IT\right]}{}^{T} & A^{\left[bridge\right]} \end{array}\right]$$

### 3.2. Model of the IT Layer Corresponding to the Communications Network

In a smart grid, the communication network is represented by a graph ($G_{C}=\left\{N^{C},\;E^{C}\right\}$), which is composed of nodes and edges. The set of nodes $N^{C}$ (comprising $n^{C}\;$nodes) is formed from the set of routers that belong to the communications infrastructure, and the connections between routers form the set of edges $E^{C}$.

**Figure 3:**
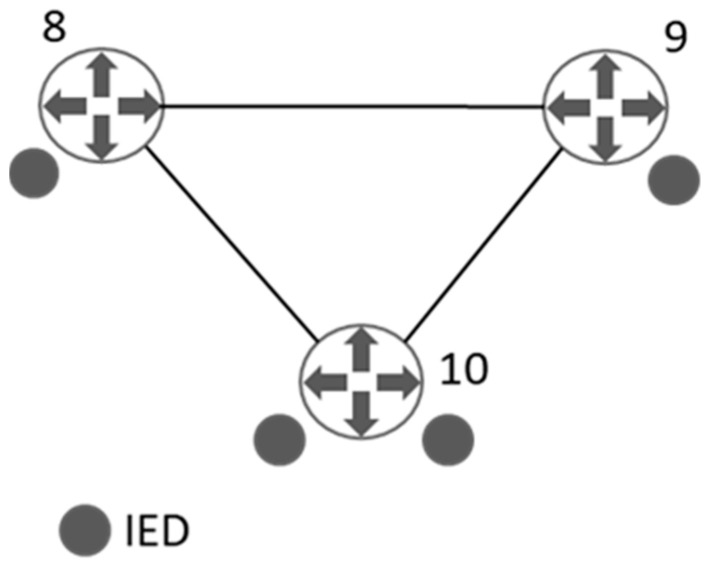
A three-buses communication network.

Figure 3 shows the communications graph corresponding to the ICT infrastructure of the smart grid displayed in Figure 2. In this case, set $N^{C}$ is composed of three nodes ($n^{C}=3$) corresponding to the three routers (labelled from 8 to 10). The three routers collect information provided by the IEDs, MUs, and controllers installed in the electrical infrastructure.

The communications graph adjacency matrix $A^{\left[C\right]}$ (26), has 3 × 3 dimensions and expresses the relationship between the elements of set$\;N^{C}$, which comprises nodes 8 to 10.

(26) $$A^{\left[C\right]}=\left[\begin{array}{ccc} a_{8\_8} & a_{8\_9} & a_{8\_10}\\ a_{9\_8} & a_{9\_9} & a_{9\_10}\\ a_{10\_8} & a_{10\_9} & a_{10\_10} \end{array}\right]$$

### 3.3. Interconnection Model between Physical Layer (OT) and Cyber Layer (IT)

As a CPS, a smart grid can be represented by a multilayer graph composed of two layers: the physical layer (OT) and the cyber layer (IT). The layers are connected by measurement and control devices installed in the OT layer, which transmit and receive information to and from the IT layer. These devices constitute the OT-IT bridge model in the coupling matrix, according to multilayer complex network theory.

In the CPS, the coupling matrix ($\hat{C}$) comprises the set of elements ($N^{P}$) in the OT layer, and their connections to the elements in the IT layer ($N^{C})\;$(27). As in the adjacency matrix, the coupling matrix element $a_{i-j}=1$ if a connection exists between nodes i and j and $a_{i-j}=0$ if there is no connection.

(27) $$\hat{C}=\left[\begin{array}{c} \begin{array}{ccc} a_{1-8} & a_{1-9} & a_{1-10}\\ a_{2-8} & a_{2-9} & a_{2-10}\\ a_{3-8} & a_{3-9} & a_{3-10} \end{array}\\ \begin{array}{ccc} a_{4-8} & a_{4-9} & a_{4-10}\\ a_{5-8} & a_{5-9} & a_{5-10}\\ a_{6-8} & a_{6-9} & a_{6-10} \end{array}\\ \begin{array}{ccc} a_{7-8} & a_{7-9} & a_{7-10} \end{array} \end{array}\right]$$

### 3.4. Cyber-Physical Smart Grid Model

A smart grid can be modelled holistically using the graph$\;G_{SG}=\left\{N^{SG},E^{SG}\right\}$, which contains the set of nodes$\;(N^{SG}$) belonging to the extended electrical graph ($N^{P}$) and the set of communications graph nodes ($N^{C})$, that is $N^{SG}=N^{P}\cup^{\;}N^{C}$s. Set $E^{SG}$ comprises the connections between all the nodes in the CPS.

The supra-adjacency matrix ($\hat{\bar{A}}$) represents the complete CPS, which comprises the extended physical layer ($G_{P}$), the cyber layer ($G_{C}$) and the interconnection between layers defined by the coupling matrix $(\hat{C}$), as observed in the following Equation (28):

(28) $$\hat{\bar{A}}=\left[\begin{array}{cc} \hat{A^{\left[P\right]}} & \hat{C}\\ {\hat{C}}^{T} & A^{\left[C\right]} \end{array}\right]$$

**Figure 4:**
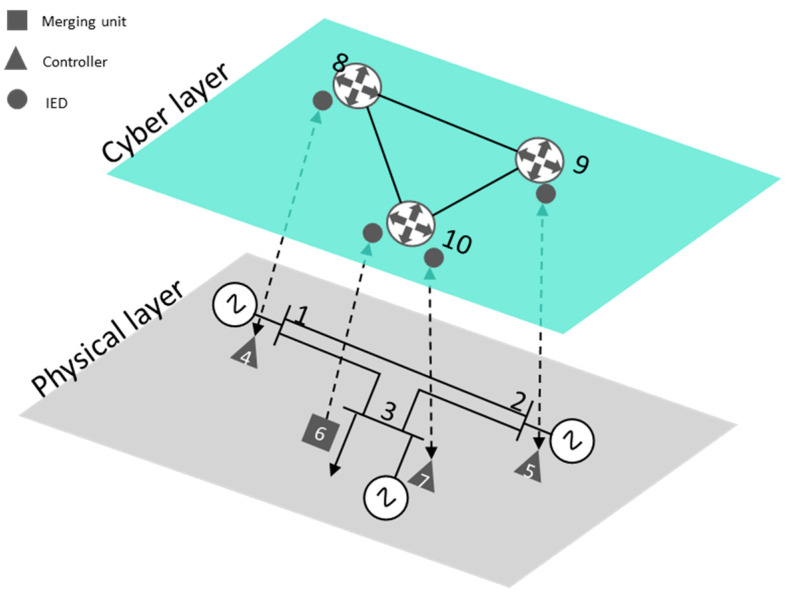
A three-buses cyber-physical network representation.

Figure 4 gives the cyber-physical structure corresponding to the three-bus network illustrated in Figure 2a. In this case, set $N^{SG}$ is composed of ten nodes, seven corresponding to the OT layer ($N^{P})\;$and three corresponding to the IT layer $\left(N^{C}\right)$. The supra-adjacency matrix has 10 × 10 dimensions, where the $A^{\left[P\right]}\;\left(25\right)$, $A^{\left[C\right]}\left(26\right)\;$and $\hat{C}$ (27) matrices correspond with the adjacency matrices of the extended physical layer, the cyber layer, and the connection between them, respectively.

Complex theory based on multilayer networks is especially suitable for analysing vulnerabilities in cyber-physical systems (composed by two heterogeneous interconnected networks) due to the fact that the methodology is very fast, and it is scalable to model heterogeneous smart grids (ITC network, power network and interdependencies between both networks). However, if the power network and ICT network have a great number of nodes and the connectivity between both networks is high, the supra-adjacency matrix employed to represent the interconnected cyber-physical system could achieve large dimensions leading to computational burden problems for analysing the smart grid vulnerability. In those situations, other techniques such as hierarchical modelling [8,11,29] can be used to reduce the complexity of the network by means of clustering algorithms which allows the mathematical dimensionality reduction. 

## 4. Vulnerability Assessment: A Case Study

**Figure 5:**
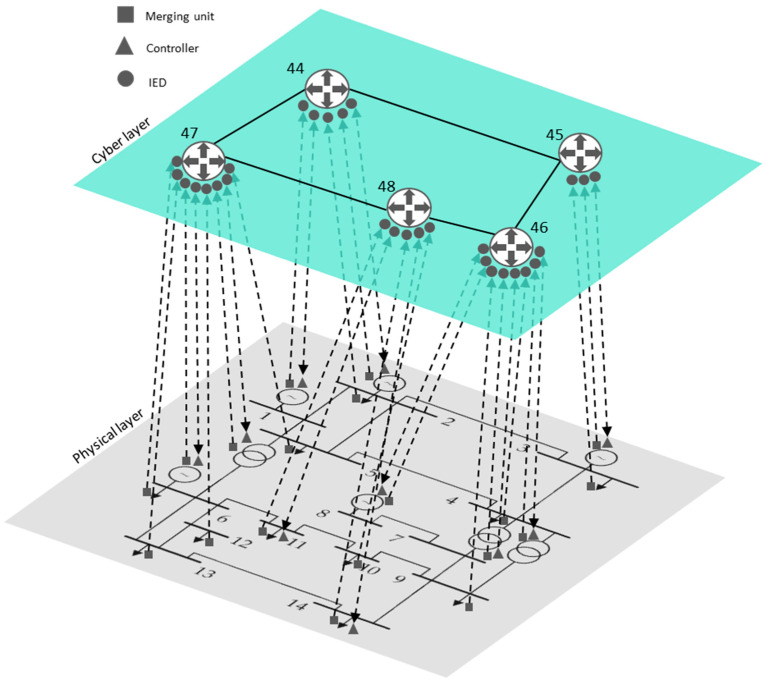
A cyber-physical representation of the modified IEEE 14-bus test case.

Using multilayer network theory, the cyber-physical model has been applied to the IEEE 14-bus test case network [30], which includes the communications infrastructure (Figure 5). The multilayer cyber-physical model consists of the electrical network layer (the grey layer in Figure 5), the communications network layer (the green layer in Figure 5) and the OT-IT interconnections between the two layers (dashed lines in Figure 5).

The IEEE 14-bus test case consists of 14 nodes, 18 lines, four generators, three transformers with regulation capacity, and 11 loads. In the cyber-physical model, each generator, transformer, or controllable load has two types of devices: a monitoring device (merging units [MUs], represented as squares in Figure 5) and a control device (controllers [C], represented as triangles in Figure 5). Non-controllable loads only have a monitoring device (MU). This case study considers the loads at nodes 11 and 14 to be fully controllable (i.e., they can receive demand response signals from the network operator).

According to the proposed multilayer network methodology (Section 3.1), the OT set $N^{B}$ is composed of 14 buses in the IEEE 14-bus test case, while the OT-IT bridge has 29 elements corresponding to monitoring and control devices located in the OT network (MUs, IEDs and controllers), which are linked to the IT layer $N^{OT/IT}$. Finally, the extended electricity network is represented by set $N^{P}$, which is composed of 43 elements.

The communications network in the IEEE 14-bus test case is modelled using five routers, which centralize the information collected from the monitoring and control equipment located in the OT layer. In this case, the five routers form a ring, as shown in Figure 5, with a total of five nodes that form the whole $N^{C}$.

In this paper, we consider the IEEE 14-bus test case as a CPS consisting of several interconnected layers. The electricity network is the physical layer (OT) and the communication network is the cyber layer (IT). Both layers are connected by elements in the OT-IT bridge. Table 2 demonstrates the relationships throughout the smart grid (electrical infrastructure, communication infrastructure and the OT-IT bridge).

In the modified IEEE 14-bus test case, supra-adjacency and Laplacian matrices have been obtained using the methodology explained in Section 2.2 and Section 2.3. The connections between elements are given in Figure 5 and Table 2.

It has to be noted that the proposed approach is effective for all attack model studies, as long as the ICT communication attacked components are included in the cyber layer and/or in the interconnection layer (cyber-power). Vulnerability assessment based on complex network can deal with random failure, natural hazard, or intentional attacks. Therefore, it is not necessary to take into consideration the attack model for evaluating the vulnerability of the interconnected cyber-physical system.

### 4.1. Cyber Physiscal System Vulnerability Analysis

The vulnerability of the cyber-physical IEEE 14-bus test case is determined using the centrality indexes defined in Section 2.4. In this section, considered events could be cyberattacks or failures in the measurement, control and communications devices in the CPS system. The CPS is composed of 48 elements: 14 physical electrical nodes, 29 OT-IT bridge nodes (MUs, controllers and IEDs), and the five routers in the cyber layer.

**Figure 6:**
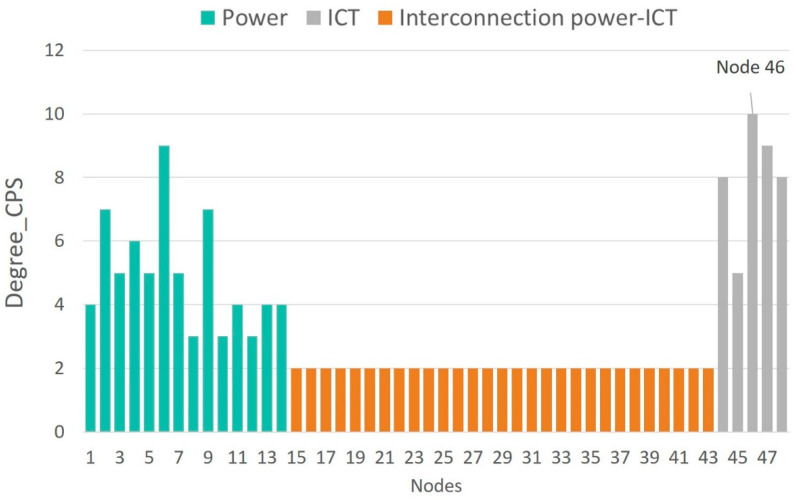
Node degree vulnerability values of the cyber-physical IEEE 14-bus test case.

**Figure 7:**
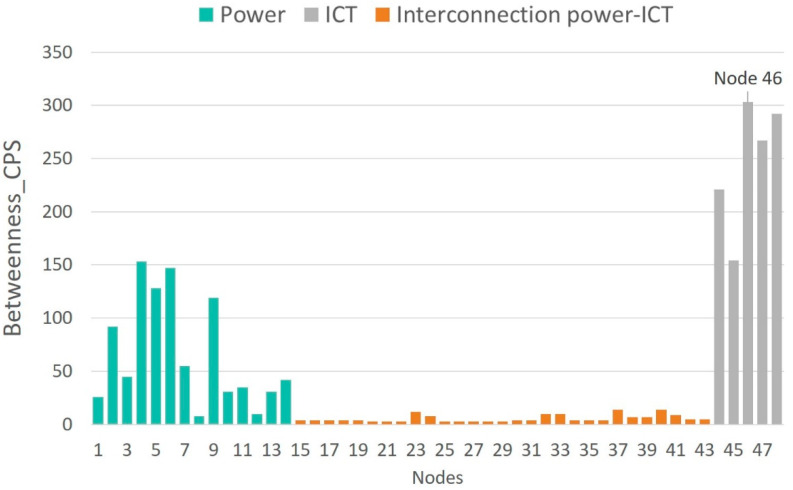
Betweenness vulnerability values of the cyber-physical IEEE 14-bus test case.

**Figure 8:**
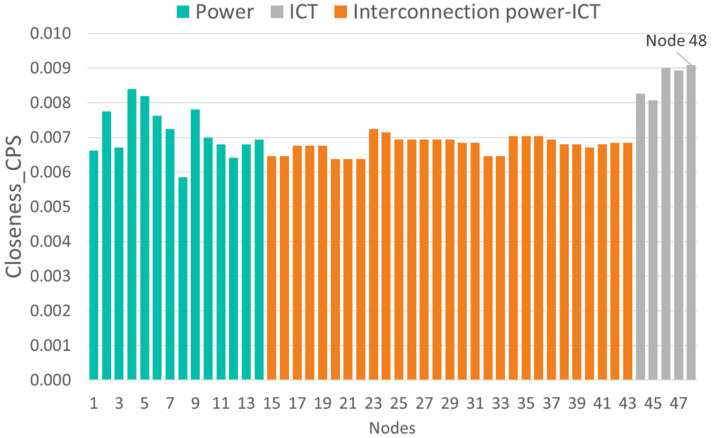
Closeness vulnerability values of the cyber-physical IEEE 14-bus test case.

**Figure 9:**
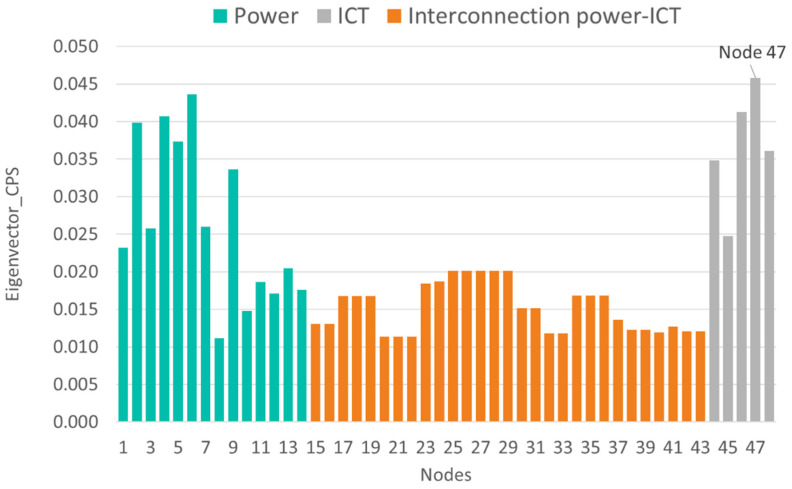
Eigenvector vulnerability values of the cyber-physical IEEE 14-bus test case.

Figure 6, Figure 7, Figure 8 and Figure 9 show the vulnerability levels of each of the 48 elements in the CPS, which are determined using the following multilayer centrality indices: node degree, closeness, between-ness, and eigenvector. Elements of the OT layer are coloured in green, elements of the OT-IT bridge are highlighted in orange, and elements of the IT network are shaded in grey. 

Analysis of the multilayer centrality indexes reveals that the most vulnerable nodes in the CPS correspond to cyber nodes (i.e., routers). Moreover, several hubs are detected in the OT layer, and it can also be noted that OT-IT bridge elements are not as critical as the OT and IT elements.

Table 3 ranks the 10 most vulnerable nodes in the CPS according to each of the centrality indexes. Nodes corresponding to the OT layer are highlighted in grey, and those representing the IT layer are coloured in green. First, it is observed that the most vulnerable elements in the CPS correspond to routers located at nodes 46 and 47 (cyber layer) and nodes 4 and 6 (physical layer).

Table 3 demonstrates that routers at nodes 46 and 47 centralize information from the IEDS, MUs and controllers located at nodes 4, 7, 8, and 9, as well as information from nodes 5, 6, 10, and 11. Hence, an attack on routers placed on nodes 46 and 47 results in a loss of information from 17 OT-IT bridge elements, such as IEDs, MUs and controllers, installed at eight electrical nodes. This analysis highlights the importance of considering smart grid networks as CPSs and not independent systems as previously discussed [3,21].

These results demonstrate that vulnerability assessments must consider the smart grid as a unique cyber-physical system rather than two independent networks. The smart-grid CPS modelled in this paper includes elements of the electrical and communications networks and considers the relationships between them.

Traditional vulnerability methods have identified vulnerabilities in electrical networks [12,13,14,15,16,17,18,19,23,24] rather than consider the network as a CPS, as demonstrated in this paper. When applying traditional methods [12,13,14,15,16,17,18,19,23,24] to the IEEE 14-bus test case, node 4 (electrical network) was found to be the most vulnerable. However, we have demonstrated that disruption to router 46 affects the information collected from electrical nodes 4, 7, 8, and 9. Therefore, a cyberattack on router 46 also represents a vulnerability in the OT network, which has not been detected by the methods described in [12,13,14,15,16,17,18,19,23,24].

### 4.2. Comparative Vulnerability Analysis

This section determines the most vulnerable nodes in the IEEE 14-bus test case using three different methods: (i) traditional OT model [12,13,14,15,16,17,18,19,23,24], (ii) IT network model, and (iii) the cyber physical system model that we propose in this paper.

Table 4 gives the most vulnerable nodes evaluated using two centrality indices: node degree index and betweenness index. The following conclusions are presented:

- Traditional OT network model [12,13,14,15,16,17,18,19,23,24]: in this case, the vulnerability analysis only covers the electrical network (nodes 1–14), the most vulnerable node is node 4, which corresponds with a substation.
- Traditional IT network model [21]: when performing the vulnerability assessment [21] on the communication network (nodes 44–48), all the nodes present the same vulnerability level, indicating that there are no critical routers that could affect the vulnerability of the IT network.
- Holistic model of the cyber-physical network: if the smart grid is considered as a CPS (nodes 1–48), it is observed that the most vulnerable nodes correspond to routers located at nodes 46 and 47. An attack that places nodes 46 and 47 out of service results in the loss of information and communication with OT nodes 4, 7, 8, and 9 and 5, 6, 10, and 11, respectively. Table 4 demonstrates that according to the node degree index in the CPS model, nodes 46 and 47 are two and five times more vulnerable compared to node 4 (in the traditional OT model) and the IT nodes, respectively. Moreover, the CPS model’s betweenness index determines that the router at node 47 has 303 critical links compared to 25 critical connections for node 4 and 1 connection for the IT nodes. Considering the betweenness index, it can be deduced that the loss of node 47 affects more than twelve times the critical connections of node 4. These results indicate that the cyber-physical network model as a whole, proposed in this paper, is the only model that allows us to identify the most vulnerable nodes in the smart grid as a single entity. Moreover, it enables us to determine the scope of a cyberattack upon routers 46 and 47 (IT) and the resulting implications for the OT layer.

Finally, the four indexes presented in Figure 6, Figure 7, Figure 8 and Figure 9 identify critical hubs in the OT network (nodes 4 and 6) and the IT network (nodes 46 and 47).

## 5. Conclusions

Vulnerability analysis in power networks has traditionally considered only the electrical infrastructure (OT). However, this representation is no longer valid in the field of smart grids, since the OT and IT heterogeneous networks are interconnected via measurement, control, protection, and communication devices.

In this article, we have proposed a new methodology to identify the most vulnerable elements of smart grids as cyber-physical systems. The smart grid has been modelled holistically using multilayer complex network theory and scale-free graphs, in which the power network (OT) is interconnected with the communication network (IT). The connection between both networks, via measurement, communication, and control devices, has also been considered. The proposed methodology is able to identify the most vulnerable elements in a smart grid CPS, which have been overlooked by traditional vulnerability methods.

Since the proposed CPS model is based on multilayer network theory and scale-free graphs, it is possible to conduct a vulnerability analysis that considers the set of elements that may experience a cyberattack, which could subsequently affect the functioning of smart grids (i.e., the communication and/or electrical infrastructure).

From the results obtained in the vulnerability analysis of traditional OT and IT networks, as well as the smart grid as a CPS, the following observations are presented:

− Traditional vulnerability assessments which focus on electrical networks do not detect those IT elements in a smart grid that anticipate a greater loss of robustness as a result of disconnection following a cyberattack.
− Multilayer centrality indices allow the detection of vulnerabilities in the smart grid as a single CPS. According to the degree index, the vulnerability of the critical node in the CPS is twice as high as the vulnerability detected using traditional methods. Regarding the betweenness index, the vulnerability of the router located at node 46 is twelve times higher than the vulnerability of the primary substation located at node 4.
− Moreover, it has been demonstrated that the multilayer centrality indices are the only indices that measure vulnerability in different heterogeneous and interconnected layers. It has been proved that routers placed at nodes 46 and 47 reveal a vulnerability in the CPS that is five times greater than the vulnerability detected in only the IT networks.
− Finally, it should be noted that holistic analysis of the smart grid reveals the existence of critical hubs in both the OT network (nodes 4) and the IT network (nodes 46 and 47).

Given these results, it can be concluded that vulnerability analysis should consider the smart grid as a cyber-physical system rather than two independent (electrical and communication) infrastructures.

## Figures and Tables

**Table 1:** Comparison table of related work with the proposed method in this paper.

		[12]	[13]	[14]	[12]	[16]	[17]	[18]	[19]	[21]	Our Proposal
**Network** **vulnerability**	**Power network**	✓	✓	✓	✓	✓	✓	✓	✓	✓	✓
	**ICT network**	✗	✓	✗	✗	✗	✗	✗	✗	✓	✓
	**ICT components**	✗	✓	✗	✗	✗	✗	✗	✗	✗	✓
	**Power-ICT** **interconnection**	✗	✗	✗	✗	✗	✗	✗	✗	✓	✓
**Complex network** **Theory**	**Multilayer network**	✗	✗	✗	✗	✗	✗	✗	✗	✗	✓
**Centrality indexes**	**Multilayer**	✗	✗	✗	✗	✗	✗	✗	✗	✗	✓

**Table 2:** Node connection in the OT (power)-IT (ICT) smart grid for the IEEE 14-bus test case.

Electrical Nodes	OT-IT Bridge Nodes (Merging Units, Controllers, and IEDs)	Routers Nodes	Electrical Nodes	OT-IT Bridge Nodes (Merging Units, Controllers, and IEDs)	Routers Nodes
1	15–16	44	8	32–33	46
2	17–19	44	9	34–36	46
3	20–22	45	10	37	47
4	23	46	11	38–39	48
5	24	47	12	40	48
6	25–29	47	13	41	48
7	30–31	46	14	42–43	48

**Table 3:** Ranked list of vulnerable cyber-physical nodes.

	Vulnerable Cyber-Physical Nodes			
	Degree Index	Closeness Index	Betweenness Index	Eigenvector Index
1	**46**	**48**	**46**	**47**
2	**6**	**46**	**48**	**6**
3	**47**	**47**	**47**	**46**
4	**44**	**4**	**44**	**4**
5	**48**	**44**	**45**	**2**
6	**2**	**5**	**4**	**5**
7	**9**	**45**	**6**	**48**
8	**4**	**9**	**5**	**44**
9	**3**	**2**	**9**	**9**
10	**5**	**6**	**2**	**7**

**Table 4:** Comparison of node vulnerability between models.

Network	Model and Node Range	Node Vulnerability	Node Degree Index Value (Node Connections)	Betweenness Index Value (Paths)
OT	Traditional power network [12,13,14,15,16,17,18,19,23,24]: {nodes: 1–14}	4	5	25
IT	Traditional communication network [21]: {nodes: 44–48}	44–48	2	1
OT-IT	Cyber physical system as a whole: {nodes: 1–48}	46, 47	10	303

## Data Availability

Not applicable.
